# Chronic fatigue syndrome/myalgic encephalomyelitis (CFS/ME) in adults: a qualitative study of perspectives from professional practice

**DOI:** 10.1186/1471-2296-11-89

**Published:** 2010-11-15

**Authors:** Simon MC Horton, Fiona Poland, Swati Kale, Maria de Lourdes Drachler, Jose Carlos de Carvalho Leite, Maggie A McArthur, Peter D Campion, Derek Pheby, Luis Nacul

**Affiliations:** 1 School of Allied Health Professions, University of East Anglia, Norwich NR4 7TJ, UK; 2 Department of Primary Care Research, University of Hull, Castle Hill Hospital, Castle Road, Hull HU16 5JP, UK; 3 Faculty of Society and Health, Buckinghamshire New University, Uxbridge Campus, 106 Oxford Road, Uxbridge, Middlesex, UB8 1NA, UK; 4 Department of Nutrition and Public Health Intervention Research, London School of Hygiene and Tropical Medicine, Keppel Street, London WC1E 7HT, UK

## Abstract

**Background:**

Chronic fatigue syndrome/myalgic encephalomyelitis (CFS/ME) can cause profound and prolonged illness and disability, and poses significant problems of uncertainty for healthcare professionals in its diagnosis and management. The aim of this qualitative study was to explore the nature of professional 'best practice' in working with people with CFS/ME.

**Methods:**

The views and experiences of health care practitioners (HCPs) were sought, who had been judged by people with CFS/ME themselves to have been particularly helpful and effective. Qualitative semi-structured interviews following a topic guide were carried out with six health care practitioners. Interviews were audio-recorded, transcribed and subject to thematic analysis.

**Results:**

Five main themes were developed: 1) Diagnosis; 2) Professional perspectives on living with CFS/ME; 3) Interventions for treatment and management; 4) Professional values and support for people with CFS/ME and their families; 5) Health professional roles and working practices. Key findings related to: the diagnostic process, especially the degree of uncertainty which may be shared by primary care physicians and patients alike; the continued denial in some quarters of the existence of CFS/ME as a condition; the variability, complexity, and serious impact of the condition on life and living; the onus on the person with CFS/ME to manage their condition, supported by HCPs; the wealth of often conflicting and confusing information on the condition and options for treatment; and the vital role of extended listening and trustful relationships with patients.

**Conclusions:**

While professional frustrations were clearly expressed about the variability of services both in primary and specialist care and continuing equivocal attitudes to CFS/ME as a condition, there were also strong positive messages for people with CFS/ME where the right services are in place. Many of the findings from these practitioners seen by their patients as helping them more effectively, accord with the existing literature identifying the particular importance of listening skills, respect and trust for establishing a therapeutic relationship which recognises key features of the patient trajectory and promotes effective person-centred management of this complex condition. These findings indicate the need to build such skills and knowledge more systematically into professional training informed by the experience of specialist services and those living with the condition.

## Background

Chronic fatigue syndrome/myalgic encephalomyelitis (CFS/ME) can cause profound and prolonged illness and disability, with wide-ranging impacts on quality of life [1] and continues to pose significant problems of uncertainty for healthcare professionals in its diagnosis and management [2]. Previous research has identified a number of problematic issues posed for health professionals, for example: the acceptance by GPs and primary care staff of CFS/ME as a legitimate condition, with many GPs remaining sceptical about its existence [3,4]; low GP confidence in making a diagnosis and managing CFS/ME [4,5]; and lack of empathy, disbelief or negative attitudes towards people with CFS/ME [6-8]. GPs who do accept CFS/ME as a recognisable clinical condition or who know someone socially with the condition have been found to have more positive attitudes [4]. The National Institute for Health and Clinical Excellence (NICE) guidelines [2] emphasize the role of primary care and the importance of working in partnership with the person who has CFS/ME [9]. Nonetheless the NICE guidelines have not been greeted with universal enthusiasm, either by people with CFS/ME or by health professionals [10]. Objections have ranged from the composition of the Guideline Development Group to the quality of evidence for recommended treatments such as Cognitive Behaviour Therapy (CBT) [11] or Graded Exercise Therapy (GET) [12]. One correspondent to BMJ Rapid Responses characterised the guidelines as "unfit for purpose" [13]; another argued that the evidence base for the Guidelines was poor [14]. A family doctor pointed out that the guidelines failed to identify the precise nature of the specialism for onward referral from primary care, asking in the light of the geographical differences in specialist care available "how do family doctors access best referral practice?" [15].

It has been argued that the role of primary care remains paramount in the treatment and management of CFS/ME because "multidisciplinary teams have not yet been set up in the United Kingdom" [9]. However, after the publication of the CFS/ME Working Group's Report to the Chief Medical Officer [16] specialist multidisciplinary teams (both in-patient and primary care-based) were set up in the UK. For example, the NHS Improvement Network (East Midlands) identifies teams in Derbyshire, Nottinghamshire, Lincolnshire and Leicestershire [17]. Such multidisciplinary teams usually consist of a co-ordinator, rehabilitation consultant or GP specialist, physiotherapist and occupational therapist, and some may have a psychologist or access to psychological services. It is claimed that 65% of the population of England is now covered by the new CFS/ME services [17].

Good communication between practitioner and patient is now recognised as a key element of good practice [2,18]. NICE guidelines state that all healthcare professionals involved in the care of people with CFS/ME should have a high standard of consultation and communication skills [2], so enabling people with CFS/ME (and their family carers) to participate in all decisions about their healthcare. Clearly, a more team-based approach should enhance communication between all healthcare professionals involved in supporting people with CFS/ME. Nonetheless, the need for better communication and education about diagnosis and management of CFS/ME has been consistently highlighted [19-21]. Despite evidence that CFS/ME should be diagnosed as early as possible to mitigate further deterioration and improve prognosis, diagnosis and the consequences of 'labelling' patients is not unproblematic, given the sometimes negative attitudes towards people identified as having CFS/ME [20,22] and the concern expressed by some GPs that giving a label of CFS/ME could actually cause harm and impair recovery [5]. Nonetheless, getting a diagnosis has been mentioned by patients as the single most helpful event for them in managing their condition [8,23]. GPs can play an important role in helping patients to understand unexplained symptoms, but while training enables them to exclude physical causes, limitations of knowledge about CFS/ME often make diagnosis uncertain, leading to frustration and impacting in turn on the quality of their relationship with the patient [9]. This may accord with the observation that doctors can struggle to maintain their professional authority when under "considerable scientific uncertainty" [20].

The medical profession is clearly not alone in supporting people with CFS/ME. Interdisciplinary and multidisciplinary rehabilitation, including peer-facilitated programmes [24] may well offer the most effective management approach to improving quality of life for people with CFS/ME. Good professional-client communication is often integral to lifestyle and educational approaches to management. Collaboration and teamwork between professionals and the person with the condition is fundamental to success [25]. Strategies which increase understanding, a sense of control and participation in treatment can have great benefit [26]. For example 42% of patients in an occupational therapy lifestyle management programme were able to return to work, voluntary work or training at 18 month follow-up [27]. This type of approach, which emphasises patient control through education, pacing and progression within a balanced programme of activity and rest, is also advocated in the management of CFS/ME-related musculoskeletal pain [28]. Goal attainment was found to be the only significant predictor of quality of life improvement in a supportive and educational group programme for people with CFS/ME [29].

The negative attitudes of health and social care professionals that have been reported may be largely explained by: well-known problems with case definition and misleading labels such as CFS, which tend to too easily aggregate a heterogeneous group of patients; the current failure of the biomedical model to explain CFS/ME; physicians' discomfort with continuing uncertainty over diagnosis and management protocols; pressure from health service funders and patients for rapid diagnosis and treatment [30]; and problems in communication between people with CFS/ME and health and social care professionals, which may lead to their further exclusion from adequate treatment or social care benefits. New and emerging scientific knowledge about the condition will continue to provide clinicians with plausible treatment strategies which can be tested in trials [28]. However, good management of the condition and its consequences for people living with CFS/ME remains a highly contended issue, exemplified in responses to the publication of the NICE guidelines.

### Aim of the present study

The aim of this study was to explore the nature of professional 'best practice' in working with people with CFS/ME. The views and experiences of health care practitioners (HCPs) were sought, who had been judged by people with CFS/ME themselves as having been particularly helpful and effective. The study therefore aims to describe, from the perspective of such HCPs, practices that: enable patients to establish the legitimacy of their condition; impact positively on the process of diagnosis and care; and enable patients to overcome experiences of social isolation and other negative effects.

## Methods

This was a qualitative study using semi-structured interviews with specialist and non-specialist health care practitioners working with patients with CFS/ME. The study was reviewed and approved by London MREC (reference 06/MRE02/58) and had PCT R&D approval.

### Participants

Potential participants were nominated by people with CFS/ME who had taken part in an associated England-wide study of their support needs (Social inequalities in the impact of living with CFS/ME: CFS/ME Observatory project). Between them, 36 people with CFS/ME nominated eight health care practitioners as having provided them with particularly helpful or effective care, based on their perceptions of the quality of care they had received. One practitioner was named by six different people with CFS/ME, and one person with CFS/ME nominated two practitioners. Of the eight practitioners then contacted by the research team, two declined to take part in the study. Participants were from the East of England and London; further participant characteristics are set out in Table 1.

**Table 1:** Participant characteristics

Specialist services	Non-specialist services	Professional groups
HCP1		
HCP2		Medicine; Occupational therapy; Physiotherapy
HCP6		
	HCP3	Medicine; Occupational Health; Holistic Practice
	HCP4	
	HCP5	

### Procedures and data collection

The eight practitioners who had been proposed were contacted, provided with information about the study and invited to participate. Six gave written consent to take part and arrangements were made for them to be interviewed individually by a member of the research team. Five interviews were conducted face-to-face at the participant's workplace, and one by telephone. Two of the authors carried out semi-structured interviews based on a topic guide (see Appendix 1).

This was developed by members of the research team, so as to reflect research literature identifying key aspects of service user and practitioner experiences of CFS/ME; provisional findings from an associated study of service user experiences (Social inequalities in the impact of living with CFS/ME: CFS/ME Observatory project); and to deploy a framework of question types (e.g. experience; opinion; feeling) [31] to organise the guide and to explore the lived experiences of the individual participants [32]. The following topics were covered in the interviews:

i) general experiences of working with people with CFS/ME;

ii) enabling people to access information and resources;

iii) recognising and responding to the needs of people with CFS/ME, including coping with uncertainty, unpredictability and stigma;

iv) enabling people to take an active role;

v) experiences of working with people from ethnic minorities, or from manual or routine occupations, or who had a severe condition.

Interviews lasted between a half to a full hour, and all (including the telephone interview) were audio-recorded.

### Data analysis

The audio-recordings were transcribed in full using English orthography, according to an agreed protocol (e.g. line numbering; speaker identification; emphatic stress in CAPS). To maintain the anonymity of the participants, transcripts were labelled simply as Health Care Practitioner numbered 1 to 6 (e.g. HCP1). Analysis followed three stages:

i) the first two transcriptions were scrutinised by two of the authors and provisional codes agreed;

ii) these codes were used as the basis for an iterative process of thematic analysis taking an interpretative stance [33] where data were organised and indexed by three of the authors using qualitative data analysis software [34] to support the process. Each of these researchers initially analysed one transcribed interview. Themes and sub-themes were identified and developed by the individual researchers and a two-stage process of cross-checking and discussion (peer triangulation) was used to validate the analysis: coding was cross-checked and the analysis subsequently refined; refined codes were then used as a basis for analysing the next transcript and these codings were cross-checked in the same way, with final cross-checking and discussion before themes and subthemes were finalised for the whole data set. The nine main themes and eighty five sub-themes which were developed initially were consolidated through further discussion and interpretive synthesis into five main themes;

iii) these themes and subthemes were then deployed in the development of materials (narrative vignettes; diagrammatic overview) used subsequently to engage with groups involved in the research (people with CFS/ME and professionals) for the purposes of participant validation. This was carried out in two researcher-facilitated workshops where feedback and comments were recorded.

### Participant validation

Two validation meetings were held. At the first in April 2009, summaries of main study themes and related samples of data were presented to twenty-three people living with CFS/ME, family carers; and ten health professionals. Findings were presented in different ways, including discussion groups, short summaries, audio recordings, diagrams and narratives over the course of a five hour event. Findings were discussed in small, facilitated mixed-constituent groups and one-to-one conversations with members of the research team and written records made. These were then reviewed by the research team. Comments of both professionals and people living with CFS/ME involved in the validation exercises showed strong accord with the findings of this study. Evidence of good face validity was provided in comments from professionals (e.g. "same stories we hear time and again, so nothing new to health professionals") and from people living with the condition (e.g. "if only all doctors were as understanding", "this confirms the need for health professionals to have the right training"). Comments which took issue with the findings focussed on the small number of professionals participating in the study; and those findings related to the demographic profile of people with CFS/ME accessing services. Comments from this event were also used to test the accessibility and perceived relevance to these groups of materials summarising the research. The presentation of study theme summaries and data extracts were revised and reorganised in the light of participants' responses to the first event. These revised materials were made available to a second group of invited participants before they attended a second, smaller, validation meeting held in October 2009 which lasted for four hours. This meeting was attended by eleven people living with CFS/ME and carers; and five health professionals. Comments from people living with the condition (e.g. "We do need to be able to trust the people who treat us"; "I would like to know that this will be seen by clinicians and other health professionals") and health professionals (e.g. "this emphasises the stigma of ME and the need to educate the public that this is a fluctuating condition and the complexity of the condition needs to be recognised") arising in discussion groups and individual conversations further confirmed the main themes of this study.

## Results

The following five themes were developed from the interview data: 1) Diagnosis; 2) Professional perspectives on living with CFS/ME; 3) Interventions for treatment and management; 4) Professional values and support for people with CFS/ME and their families; 5) Health professional roles and working practices. These are set out in Table 2 below, which displays each main theme with associated subthemes and illustrative quotes from participants. These themes and incorporated subthemes are set out in the following sections.

**Table 2:** Main themes, subthemes and illustrative quotes from interviews with health care practitioners (HCPs)

Main theme	Associated sub-themes	Illustrative quotes
Diagnosis	*The process of diagnosis*	"I think there's still some doubt you know amongst quite a lot of professionals as to what, what the condition is ... in fact I think whether it exists at all" (HCP3)
	*Professional experience and expert practice*	"... the more people you come across the more you pick up these little differences all round the edges ... because they kind of sound very significant actually there's variability and the differences 'cause often there's a set range of symptoms that we use to diagnose..." (HCP2)
	*No diagnosis, delayed diagnosis, or misdiagnosis*	"I think most people by the time they've got to us they've been round the houses so many times or they've seen other consultants or they've been to the GP backwards and forwards" (HCP1)
	*Confirming the diagnosis*	"I think there's always some I suppose that we would say are sort of idiopathic chronic ... they perhaps appear to have all the kind of signs and symptoms but maybe they still don't they don't quite fit the criteria..." (HCP1)
Professional perspectives on the impact of living with CFS/ME	*Accepting the diagnosis*	"I think they find it difficult to accept that they've got anything wrong with them and if they have got something wrong with them that anybody else will believe that there's anything wrong with them" (HCP3)
	*The impact on life and living*	"there's lots of activity that they don't even acknowledge they're doing just because it's such basic day to day stuff ... they'll only think about the big things but um it isn't just the big things" (HCP2)
	*Families & family burden*	"carers find it quite difficult to know how to help and they might give very mixed messages sometimes 'cause they don't know whether they should be telling them to get off their bottom and get on with it or whether they should be telling them to rest" (HCP2)
	*Financial burden*	"people get incredibly stressed about their benefits" (HCP2) "some people obviously financially have to work even if maybe it's not the best thing for their health" (HCP1)
Treatment and management	*Initial stages*	"I think those people who come in with the kind of mind of 'you're going to kind of give me information and advice and I'm going to go away and put it in place' those people do really well. I think those people who come in and want you to do something to them and make it better I think inevitably those people are disappointed because we can't do that" (HCP1)
	*Complexity and severity of the condition*	"... everybody's different and you know people haven't always got one thing...um they might have other problems as well, so then it starts to get even more complicated because they're supposed to be doing one thing for one medical condition but the fact that they've got their ME means they're struggling to do what they should be doing for the other one..." (HCP2)''
	*Progress and change*	"she slowly moved forwards with initially hardly being able to do anything at all to now she's going out on a daily basis, now this is an illness that is spread over seven years, eight years, and she is now improving, we also had another patient in there who, who improved completely and has gone back to work" (HCP6)
	*Alternative treatment approaches *&* the evidence base*	"...we see people who've tried all sorts of different things and some of those things are useful ...or those people feel like they're useful but it it's very it's difficult for our point of view because there's no sort of evidence base for any of those things..." (HCP1) "We have different types of treatments looking after them, like acupuncture, herbal remedy, tonics, and life style. I give them a guide line to say you should do that and try not do that and diet wise we give them suggestions, certain diet is good for this condition but something else are not too good" (HCP5)
	*Cognitive, psychological & counselling approaches*	"some people ... perhaps maybe don't need that kind of formal input and maybe just access counselling or they use the kind of link worker service via their local GP surgery um again just as a sort of means of more support ... that works well for some people depending on what the issues are - some people don't need a kind of a whole formal sort of CBT process some people just need some simple counselling..." (HCP1) "...if you push yourself and you get tired and you get symptoms then ... some people will tend to remain within the comfort zone, that's not the natural reaction, those are the people who actually do the best from CBT because it actually helps them to break that cycle..." (HCP6)
Professional values and support for people with CFS/ME and their families	*The existence of CFS/ME as a condition*	"I would say probably half of general practitioners don't understand it and regard it as a psychological manifestation, rather than a physical [one]" (HCP6) "you sometimes get whole practices which decide ME doesn't exist" (HCP6) "we do have patients who see GPs who will quite happily say to them they don't believe that CFS exists" (HCP1)
	*Therapeutic relationships and client centeredness*	"I think most people go into their GP with a symptom that's really worrying them at that point or something they really need help with but they probably don't tell them the whole because they've got five, ten minutes" (HCP1) "with this particular condition they have to keep having help in different time periods - it means what I did for her she could have been better for six month and a little stress or something, symptoms came back so she's back" (HCP5)
	*Information available for people with CFS/ME and others*	"... there is quite a lot of other stuff out there ... nowadays it's very easy for people to just type it into the internet and all sorts of stuff comes up ... that is very confusing for people - you know there's lots of people offering all kinds of magic wonderful answers and I think when you want to get better it's very easy to believe all those things so I suppose we're quite careful about where we direct people to..." (HCP1)
	*Supporting people with CFS/ME in the workplace*	"..the kind of return to work programs that they are used to doing with people who have broken their leg its completely different in that you can't get someone back to work with a condition like this in six weeks and they are not going to go from no hours to full time in a short space of time". (HCP1)
Health professional roles and working practices	*Enabling self-management*	"I do advise them very basically and very basic things on the boom and bust here in terms of the charging the battery and not to spend it immediately, that they will have good days and bad days and, and again to actually have a constant energy expenditure on good and bad days" (HCP4) "if you think how people normally deal with an illness if you get the flu you go to bed for a week don't you and then as it goes away you start to get up and you just get going and you do a bit more every day 'till you get back to normal and that's a normal response we all understand to illness, and so people think they can do the same thing with this but it doesn't work quite the same" (HCP2)
	*Specialist services & working partnerships*	"...certainly sometimes people have to push to get referrals to a clinic like ours um or quite often we see people who've they've been on the internet and they've found us and given the GP the details and then the GPs made a referral" (HCP1) "it's never going to just affect that one person in isolation they have to have that support set up around them so most people we see we would see with someone else like that's often and again because most people's maybe concentration isn't very good they often need someone else to remember all the information" (HCP1)
	*Diverse populations*	"..yeah I think we very much have a kind of ninety ninety-five percent of our case load is a very kind of standard set of people and we really don't have very many people from kind of lower socio-economic groups or from ethnic minorities - a few but probably even the people we have from ethnic minorities probably still fall into that very kind of middle class group...we don't have many people who fall outside of that it's very unusual and [this is] a very ethnically diverse place" (HCP1) "what I've seen over time is the middle class turning up in my clinic because they want an explanation" (HCP6)
	*Professional frustrations*	"I feel a bit frustrated if people need help and probably society doesn't realise they are ill - I do have a patient with ME who said my GP said it's in your mind, I don't believe she has something in her mind, she's depressed and she's very tired she struggled to get up but probably even family members and friends don't understand, they think you are lazy" (HCP5) "some people never get past that and so they're very stuck uh so it's quite difficult to get in there unless you've got that cooperation and the willingness to work together" (HCP2)

### 1. Diagnosis

It was generally acknowledged that reaching a firm diagnosis of CFS/ME can be challenging for GPs working in primary care, who may have little experience of the condition. Even though several participants thought that the NICE guidelines were proving helpful, they saw the lack of any diagnostic test giving conclusive proof of the condition as impacting on practitioners and patients alike. One view was that until such a test is developed the existence of the condition will remain in doubt amongst some medical practitioners and policy-makers; patients on the other hand may continue to demand more investigations to try and prove the authenticity of their condition. However, the process of diagnosis in primary care was thought to have improved in recent times: becoming more timely, with earlier referral to specialist services, more certain and more accurate.

Exposure to new presentations of CFS/ME was considered important for improving primary care practice. It enabled practitioners to recognise the condition and develop confidence in their diagnostic skills. Very careful case history-taking, listening carefully and patiently to presentation of symptoms, with appropriate investigation were all considered vital elements of practice. It was acknowledged that some people with CFS/ME had had "quite a rough time with the medical profession" (HCP2) or worse still, "had been terrified by their previous contacts with medical services" (HCP6). Specialist practitioners develop awareness of the wide range of symptoms, whether physical or psychological, that can be associated with the condition, and their significance, through extensive exposure to CFS/ME.

The negative impact of 'no diagnosis', a delayed diagnosis or a mis-diagnosis were clearly acknowledged by these participants. Consequences of delayed diagnosis for improvement and recovery were considered significant, acknowledging that this left patients uncertain and with entrenched and often unhelpful patterns of behaviour. Misdiagnoses which were mentioned by these participants included depression and treatment for depression. One respondent talked about a woman who had been on antidepressants for two years and described her as feeling, "emotionally numb but not physically any better" (HCP3). One practitioner spoke about a patient who had been told by her GP, "it's in your mind" (HCP5) and pointed out that she was clearly depressed because she was tired, struggling to get up, and facing disbelief even from family members and friends. They realised that there are still patients whose diagnosis remains uncertain and where practitioners face the continuing challenges of unravelling conflicting evidence and beliefs. They affirmed that confirmation of diagnosis may represent the end of a long period of uncertainty for a person with CFS/ME and may thus be a significant relief: "they know what's wrong with them and they have an idea of what to do" (HCP1). However, they also observed that for someone who may have only been experiencing symptoms for a short period of time, the presentation of this diagnosis may not be quite so welcome.

### 2. Professional perspectives on the impact of living with CFS/ME

These practitioners felt that despite the periods of great uncertainty, doubt in themselves and growing mistrust of the medical profession, experienced by people living with CFS/ME, they may still find it very hard to accept the ultimate diagnosis. They highlighted how negotiating such ambivalence on the part of their patients could in turn create particular challenges for the health professionals engaged in imparting the diagnosis and supporting patients in managing its consequences. Adjusting to and accepting a long-term illness and its disabling consequences was acknowledged as being extremely testing, whatever walk of life a person may be from; yet people living with that illness may also be faced with the doubts of others about the authenticity of their condition. Practitioners found that while some people will accept that this is a long-term condition which may entail slow progress towards recovery, others will struggle to accept and then adjust or even to acknowledge that they have made any progress. People may go through a grieving process and experience bereavement for the things they have had to let go; this often takes time to work through and deal with, all issues which may call for sensitive support from the health practitioner.

Specialist practitioners found they therefore needed to provide holistic and flexible support for patients faced with managing serious restrictions to their lives and lifestyles which may lead them to stop work, and in extreme cases to become bed- or housebound for long periods. At the very least they have to constantly manage their activity levels so as not to push themselves into over-activity which can trigger the 'boom and bust' experience described by most of these practitioners, and being forced to remain in bed for several days to recover. These practitioners felt that people with CFS/ME may be stigmatised in the workplace, pressurised into downplaying their symptoms; living in fear both of losing what they have and of having a relapse due to consequent overactivity.

Specialist practitioners highlighted that CFS/ME impacts not only on the 'big things' such as employment, but even on basic day-to-day leisure activities such as reading or watching TV, which need concentration and entail a significant memory load. People's ability to sustain a conversation and take in information may also be impaired, with implications for how intervention is managed by healthcare practitioners. Even very simple things can cause a great deal of stress: not being able to get the children to school, getting into difficulties with the Local Education Authority, and consequently unable to do anything else until such ramifications are all resolved.

These practitioners identified that such dilemmas can further complicate access to support. Perhaps because of fears that family, friends or colleagues will not understand the condition, people with CFS/ME may try and keep other people, including members of their support network, away when they are at a low ebb. This can also prevent them really understanding the seriousness of the condition. People who do have good support tend to do better, but some people's lives and commitments make it very hard for them to act on advice. Parents of people seriously affected by CFS/ME may come under extreme pressure to have them back at home. It can be very difficult for carers to know how to act for the best, and they may end up giving very mixed messages, which may be confusing.

Financial pressures were identified as having a significant negative impact on health and recovery for people with CFS/ME. Personal and workplace pressures may be exacerbated by negative or inflexible responses from agencies involved in assessing eligibility for incapacity benefit (although there was some praise for doctors who conducted the medical assessments entailed here), potentially causing additional stress. People with CFS/ME may also end up paying for various types of private treatment, such as holistic or alternative treatments, but also CBT. Again, this makes access to health practitioner support less straightforward and more complex.

### 3. Treatment and management

Practitioners from specialist services felt that initially, receiving a diagnosis of CFS/ME may be so overwhelming that some people are unable to contemplate the implications or take anything in. Others, however, are well-prepared and well-informed, expecting to manage the condition themselves and implement changes to their lives and lifestyles. Those who do so tend to do well, whereas those who expect "you to do something to them and make it better" (HCP1) are inevitably disappointed and may not do well. They saw these patients as needing time to adjust to the necessity of making life changes; but even so, some may carry on, "fighting the idea" (HCP2) of CFS/ME.

The vast majority of people seen by the HCPs from specialist services were described as being 'up and about', able to attend a clinic and hold conversations. However, participants emphasised the variability between patients presenting with symptoms apart from the fatigue and where some other symptoms, such as headaches, gut symptoms or muscle pain may be predominant for some individuals. Additional complications arise with co-occurring medical conditions where, "the fact that they've got their ME means they're struggling to do what they should be doing" (HCP2). A very small proportion of people seen by specialist HCPs were living with a severe condition and were significantly unwell, confined to home, or bedbound in a darkened room, unable to communicate. Even the specialist HCPs found this extremely challenging and they may have very few helpful suggestions. Specialist HCPs would visit people with a serious condition at home, or if appropriate maintain contact by phone, especially to offer support for the family. People who had been living with a serious condition for a long period (e.g. 7 to 10 years) were particularly vulnerable due, for example, to the complex psychological issues associated with making the transition from childhood to adulthood while socially isolated and set apart from their peer group.

For this, admittedly minority, group these practitioners saw progress and change for the better as fraught with difficulty. They saw some of these patients as not knowing how to move forward, how to recognise or acknowledge improvement, even when this seemed obvious to the practitioner, and that their experience might well be enhanced with highly specialist psychological support, which, however, practitioners may not be able to access on their behalf. For other patients, even those with a severe condition, the messages tended to be positive - that "most people do come out in the end and they do start to move forwards" (HCP1).

These specialist practitioners recognised that for those with long-term illness, changing established patterns can be very hard. Some did report success stories, such as examples of people making a full recovery and returning to work, as well as examples of relapse and the breakdown of family support systems. While they thought that most of such individuals coped, "incredibly well" (HCP1) progress in itself can be difficult. Some people continue to fight the idea of CFS/ME and its implications, including actively seeking to engage with health professional services. It may take many months before they accept the condition and decide to make positive steps to change their lives by giving up work, reducing working hours, and making significant lifestyle changes. Then they may return to a specialist HCP for support. However, it is often hard for people to see that they have made progress, and progress when it comes may never be quick enough, in turn placing understandable pressure of expectations on the practitioner-patient relationship.

People try all sorts of alternative treatments and therapies, including diets, acupuncture, herbal remedies, tonics and lifestyle interventions. While some of these are reported as helpful, or at least found helpful by people with CFS/ME, from an NHS practitioner's point of view making any recommendations of such treatments is difficult because there is no evidence base for them.

Aspects of cognitive behavioural therapy (CBT) can be very useful in helping people break counterproductive patterns of thought and behaviour in some cases. However, engaging in the whole formal CBT process may not be necessary. Specialist practitioners were of the view that, in fact, for those who are very poorly, it is pointless. The stage at which CBT is brought in is important and relevant, but for many, the sort of counselling service available through a GP practice may be enough. Specialist HCPs said they often used CBT principles in their practice, especially where unhelpful patterns of thought and behaviour, anxiety or stress were evident. NHS HCPs all emphasised how difficult it was for adults with CFS/ME to access formal CBT, despite there being a small proportion of patients who would definitely benefit. Adults with CFS/ME rarely met the strict acceptance criteria set by NHS mental health services for CBT.

### 4. Professional values and support for people with CFS/ME and their families

Specialist HCPs identified a core minority group of GPs in their region who made referrals to their service, but contrasted these GPs with the many who did not understand CFS/ME, and who see it as a psychological rather than a physical condition. They reported whole practices as having decided that CFS/ME did not exist and that many GPs would never make a referral to a specialist service. Participant HCPs reported how some patients told them that their GP openly stated their lack of belief in the existence of CFS/ME.

All participants emphasised the importance and powerful therapeutic value of listening. One specialist said that, "patients will often say 'you're the first people that have actually listened to me'" (HCP2). Time limits in the primary care system often constrain patients from recounting their full story, and, "the doctor is too busy, they don't have time really facing the patients for five minutes just to say OK, then the computer, then done" (HCP5). However, these participants reiterated the need for practitioners to be knowledgeable, empathic, inventive and capable of learning, acknowledging the patient's condition and taking it seriously. They should be able to respond flexibly to people's needs, accommodate the difficulties inherent in the condition that affect concentration and/or physical access, remain positive and encouraging and work in ways that engender a trusting relationship.

Trust was considered to be a primary issue. For example, specialist practitioners were very clear in saying that not all sources of information were to be trusted. For some people with CFS/ME the internet may provide valuable information on specialist services; for others, it may be hard to access and a source of confusing and misleading information, "offering all kinds of magical, wonderful answers" (HCP1). Practitioners therefore reported exercising care about where they direct people for information which will help them understand or explain their condition to others; HCPs from specialist services reported using standard information packs and DVDs, and directing people to local support groups or expert patient programmes. They also reported recommending leaflets produced by *Action for ME *or the *ME Association*, and referring people to the *Citizen's Advice Bureau *(CAB) or *Disability Information and Advice Line *services (DIAL UK) for advice on disability-related support matters such as benefits or mobility issues. They highlighted the importance of providing appropriate and accurate information for employers of people with CFS/ME as well as employees with the condition. It could take pressure off the employee by making it clear that, "recovery [would] be a matter of months rather than weeks" (HCP4). One specialist practitioner felt that people with CFS/ME were stigmatised when it came to the workplace and advised them to, "tell their employer that you've just got post viral fatigue and it will get better, don't tell them you've got low grade ME" (HCP6). Specialist HCPs would often see people from high-stress, highly demanding jobs but felt it was unusual to get them back into the same type of job. They would often choose not to return to the kind of work that might have been part of the problem. However, some people might not have the choice, and would have to continue in full-time employment, limiting the potential for the practitioner to support life and work change strategies. Participants found that patients who were not in work mostly wanted to get back to employment, but that they needed to be supported to establish a consistent pattern of activity as an essential precursor to any successful return.

### 5. Health professional roles and working practices

Specialist HCPs all focused on the importance of engaging patients and enabling them to manage their own condition. Succeeding in doing this depended on their developing a relationship of trust, where they could support people to think things through, and then make their own decisions based on a clear understanding of what might need to be changed. Specialist HCPs described using a number of skills to enable patients to gain clarity and insight. They encouraged people with CFS/ME to manage their overall activity in different ways, rather than always just to reduce their activity. To develop a constant and consistent expenditure of energy, people with CFS/ME needed to understand their symptoms but it was not helpful for them to become too symptom-focused. These practitioners recognised that the challenges for people with CFS/ME were often highly complex and not to be underestimated.

Specialist HCPs acknowledged how much pressure some people had had to exert just to get a referral to their service. They emphasised that there was a need for specialist services to be more 'visible' and to provide education for other HCPs, GPs especially, because, "there is quite a lot of ignorance about the condition in the GP population" (HCP3). This perhaps was understandable because GPs lacked frequent exposure to these patients. Specialists had both experience and expertise to be able to support GPs and other HCPs in reaching or confirming a diagnosis (e.g. post viral fatigue vs CFS/ME), giving advice on appropriate medication, or providing services such as specialist Occupational Therapy. Specialists were involved in supporting people applying for benefits, often trying to help other agencies understand the variability inherent in the condition. They reported working in flexible ways with families or partners as well as with the person with CFS/ME themselves. This is partly because the family is naturally implicated due to the nature of the condition in restricting many everyday activities, but also because the person themselves may be unable to take in information and advice. They did indicate that referrals to the specialist service from GPs who did refer were becoming more timely.

Most of the specialist HCP caseload was reported to be white middle class and female, despite the participants' services being located in an ethnically diverse area. Patients seen were mostly in the twenty to thirty age group, far fewer men than women and far fewer people from manual or routine occupations, again despite the demographic structure of the catchment areas. One specialist HCP thought that the people from ethnic minorities who were seen in their service tended to be middle class anyway, and that men in particular, found it hard to come to terms with the loss of role associated with giving up work. The one exception was the HCP from London who was a holistic practitioner and who saw a range of nationalities: "...Pakistani, Indian, English local...no huge difference [in treating people from different ethnic groups]" (HCP5).

All HCPs who were interviewed described several sources of professional frustration in the course of working with people with CFS/ME. These included the lack of recognition or common acknowledgement of the condition by society and its institutions, such as health or benefits agencies; poor access to resources such as CBT or other psychological services when they were thought to be necessary; people with CFS/ME themselves, who either refused to acknowledge progress or ignored the advice that had been given but came back again and again; and finally, feelings of inadequacy due to the general limitations of knowledge and understanding about the condition and how to help people who are stuck and not making progress.

## Discussion

This study has explored the views and experiences of a small number of healthcare professionals who were considered by people with CFS/ME to have been particularly helpful and effective in their management and support. Despite the small number of participants in the study the amount and type of data collection was consistent with qualitative studies of this type. By accessing participants with varying levels of experience and expertise this study was able to explore issues through a range of viewpoints [32], capitalising on the unique circumstances of the individual participants but open to the recurrence of shared issues and insights [35].

It is perhaps worth more than passing consideration that from a group of 36 people with CFS/ME only eight practitioners were nominated on the grounds of helpful and effective management and support. Findings from these interviews reflect practitioner experiences over a number of years, covering periods before and after publication of the NICE guidelines, but before the introduction of 'Employment and Support Allowance' and the associated 'Work Capability Assessment' by the U.K. Government. Some comments, however, specifically relate to the period following publication of the NICE guidelines in 2007. The findings highlight key themes which reflect a clear and sensitive recognition by these practitioners of the experience of living with CFS/ME, and the sometimes complex challenges to delivering 'best practice'. Such challenges certainly include the frequent and marked uncertainty which may be shared by primary care physicians and patients alike, and those also associated with being given a diagnosis of CFS/ME; the continued denial in some professional quarters of the existence of CFS/ME as a condition; the variability, complexity, and serious impact of the condition on life and living, the 'big' issues such as employment and finance and also routine activity; the onus on the person with CFS/ME to manage their condition, which can and should be eased with support from HCPs, and the need for appropriate and timely specialist intervention in some circumstances; the wealth of often conflicting and confusing information on the condition, options for treatment; the vital role of extended listening and trustful relationships with patients and the need to actively 'co-ordinate' the patchwork of support potentially available to them.

The scepticism of some GPs that CFS/ME actually exists [3,5] is still seen to be an ongoing and complicating issue. While the introduction of the NICE guidelines was reported to have improved the efficiency of onward referrals to specialists, there was clearly a well-founded perception that for people presenting with symptoms in primary care management of the condition was still a lottery. Practitioners in this study thought such inequity had an impact not only on a patient's current well-being but also on their prognosis. GPs accepting CFS/ME as a clinical entity tend to have more positive attitudes to this group of patients [4], but there are clearly still GPs whose lack of confidence in diagnosis, views on the legitimacy of the condition and attitudes to onward referral to specialist services reflect those of studies elsewhere [5]. Positive attitude, encouragement and ongoing support were linked with successful outcomes by participants in this study. These practices align well with the general principles of good patient-centred communication for building trustful relationships [26] and the expressed needs of people living with CFS/ME [8].

Being given a firm diagnosis of CFS/ME could clearly be just the start of a difficult struggle to get well for many people with CFS/ME, according to the specialist practitioners in this study. They underlined the complexity of the condition in terms of health and psychosocial impacts, which were challenging not only for patients but also for HCPs supporting them. Wider scepticism about their condition and conduct [7] was encountered within the family, the workplace and in dealings with statutory services such as benefits agencies. Flexible attitudes and ongoing support was considered an essential part of specialist HCP working practice, as emphasised in the NICE guidelines [2], but working in these ways was also described as challenging, not only in the health and social care context, but also within the context of the client-practitioner relationships, especially where clients expected speedy treatment and recovery [30].

A self-management approach was clearly advocated by all participants in the study for an otherwise often-disempowered patient group. Self-management programmes [24] which include strategies such as client-centred goal setting, self advocacy training, education and peer mentoring encouraged cautious optimism among participants for improvements in quality of life of people living with CFS/ME. The impression from specialist HCPs in this study was that flexibility and responsiveness, combined with additional support from peers or peer groups and third sector organisations contributed to a necessarily patchworked set of approaches. These practitioners did not consider interventions such as CBT as simple panaceas for all people living with the condition, but to be implemented where appropriate, and working effectively if well-targeted. There was a clear need expressed by practitioners for more knowledge on the part of their colleagues about the condition and about interventions for the condition, including CBT. The need for more research into specifically how CBT may contribute to physical improvement and which elements of it are essential to recovery has been underlined [18].

The positive message that most people do well - eventually - echoes the single case study of a young woman with a severe condition [25]. The authors emphasise the challenges faced by client and practitioners alike, stress the vital role of teamwork in addressing the complexities of CFS/ME, as well as the problems and pitfalls facing patients after a lengthy illness, and that "a fear of progress is real for some patients" [25]. According to HCPs in our study, located across rural, semi-rural and urban populations, teamwork happens pragmatically across a distributed network of locations, services and individuals. Providing information and keeping knowledge about specialist services up-to-date may be hard to achieve, but was clearly a key means for developing and sustaining quality provision for people presenting with symptoms or living with CFS/ME. It has been argued [9] that people with CFS/ME themselves could also be a valuable resource in interactive educational initiatives for family doctors' training and Continuing Professional Development (CPD), and that such initiatives might counter the evident lack of preparation for primary care management of this condition. Training of medical and other health professionals clearly needs to address not only shortfalls in knowledge but also the significant attitudinal barriers that clearly still abound [8,21].

The validation process, with careful attention to presentation and discussion of findings with relevant groups generally gave credence to the study findings. However, one of the workshop delegates commented that the study participants' assertion that the majority of people they saw were white, middle class and female, did not accord with their own experience. The findings from this small qualitative study of HCPs' experiences clearly do not permit generalisation on this and other counts. Nonetheless the sample includes participants from a specialist team which covers urban and rural areas in a region with a widely ranging socio-demographic profile, suggesting that even in such a context individuals from other groups may find accessing specialist and/or constructive care to be problematic.

## Conclusion

This study has reported on the views and experiences of a small number of health professionals from a range of disciplines, who were judged by people with CFS/ME to have been particularly helpful and effective in their management and support. Professional frustrations were clearly expressed about the variability of services both in primary and specialist care and continuing equivocal attitudes of some professional colleagues to CFS/ME as a condition and which could impact both on timely diagnosis and on consequent timely access to other support. However, there were also strong positive messages for people with CFS/ME, where the right services are in place. Many of the findings from these practitioners seen by their patients as helping them more effectively, accord with the existing literature both from the practitioner and patient perspective identifying the particular importance of listening skills, respect and trust for establishing a therapeutic relationship which recognises key features of the patient trajectory and promotes effective person-centred management of this complex condition. The findings of this study indicate the need to build such skills and knowledge more systematically into professional training informed by the experience of specialist services and those living with the condition.

## Appendix 1

Interview topic guide

1. What is it like in your experience to work with people with CFS/ME? Perhaps you could think of a client who you have worked with and tell me what you did.

2. If I was someone with CFS/ME, how would you help me get the information and help I needed?

3. How well do you think you recognise and respond to the needs of people with CFS/ME?

4. There is often a good deal of uncertainty or unpredictablity in people's CFS/ME symptoms over time - can you think of ways in which you have helped people deal with this?

5. Think about someone with CFS/ME who has experienced stigma (negative attitudes) - tell me what happened and why it came about.

6. There is a great deal of emphasis these days on clients taking an active role in their health/social care - how do you understand this client role in working with people with CFS/ME?

7. There are people from some socio-economic groups, ethnic minorities or who have a severe condition who may have especial difficulties in accessing services - have you had experience of working with any of these groups? Can you describe your experiences?

8. In your experience, what training is needed for professionals working in your field?

## Competing interests

The authors declare that they have no competing interests.

## Authors' contributions

MdeLD, JCdeCL, SH, MM, DP, LN and FP were responsible for conception of the study; MM, FP, MdeLD, JCdeCL, DP and LN were responsible for the ethics application (REC 06/MRE02/58); MdeLD, JCdeCL, SH, FP and MM designed the methodology; SH, FP, MdeLD, JCdeCL, MM, PC and DP developed the topic guide; SH and JCdeCL conducted the interviews; SH, MdeLD, SK and FP carried out the data analysis; MM, FP and SH organised the validation exercises and dissemination events; SH, FP and SK organised the writing up of the article; SH, SK and FP wrote the first draft of the manuscript; DP, LN, and PC commented on the manuscript and all authors approved the final version.

## Pre-publication history

The pre-publication history for this paper can be accessed here:

http://www.biomedcentral.com/1471-2296/11/89/prepub
